# Case of Successful Transfusion Resorted to for Severe Hemorrhage after Delivery

**Published:** 1878-03-20

**Authors:** Robert Cory, Charles Cameron

**Affiliations:** Assistant Obstetric Physician to St. Thomas’ Hospital; Resident Accoucheur to the same hospital


### CASE OF SUCCESSFUL TRANSFUSION RESORTED TO FOR SEVERE HEMORRHAGE AFTER DELIVERY.

By Robert Cory, Assistant Obstetric Physician to St. Thomas' Hospital; and Charles Cameron, Resident Accoucheur to the same hospital.

On the afternoon of June 26th, Mr. Cameron was called to Mrs. S. L——, on account of severe hemorrhage after delivery. She was thirty-four years of age, and had not previously borne children. It was ascertained that she had been losing blood for fourteen days previous to the above date, that the labor had been natural, and that the child, which was living, had been born one hour and a half before his arrival. He found that hemorrhage was still going on, and that the placenta was adherent to the fundus and anterior surface of the uterus. The woman was in a state of collapse, the pulse was scarcely perceptible at the wrists, the face and lips were blanched, and the extremities cold. Mr. Cameron removed the placenta as quickly as possible, but much blood was lost while this was being done, and the uterus did not contract afterwards. Some liquor ferri perchloridi fortior diluted (1 vol. to 4 vols. of water) was accordingly at once injected. This immediately arrested the hemorrhage. Hot-water bottles were then applied to the feet, two brandy enemata injected, and Dr. Cory sent for to perform transfusion.

On seeing the patient, Dr. Cory determined at once to have recourse to the operation. The woman was now quite unconscious, and kept throwing her arms from side to side. Dr. Roussel's apparatus was at hand at the hospital. This was obtained without delay, and Mr.

Cameron offered his blood. The cup of the instrument was applied over his left radial vein; then the left radial vein of the woman was opened, but this was found too small to admit the cannula for a sufficient distance without splitting. Accordingly the right median basilic was substituted; this was of ample size. When all was ready, Mr. Cameron's vein was pricked. The blood flowed readily, and filled the injecting ball rapidly, so that in a very short time ten ounces of blood had passed. At this point the injecting ball began to leak,* and as it was feared air might thus be admitted, the operation was discontinued. Immediately on the passage of the blood the woman seemed to revive, and at the close of the operation was evidently better. The color had returned to her face and lips, and the pulse, before imperceptible, was now evident. On leaving the house half an hour after this, the woman seemed much better, but not long afterwards Mr. Cameron was again sent for, and found her with livid face, restless and collapsed, although there had been no fresh hemorrhage. Brandy was administered by the mouth, and hot-water bottles kept to the feet.

On June 27th, at 1 a.m., her extremities were warm, she was perfectly conscious, put her tongue out when asked, and said she had no pain except in her head. Temperature 37.8° C. Taking iced milk and brandy as nourishment. Respiration normal. At 11 a.m. she spoke, and seemed quite rational; said, in answer to questions, that she was 34, and had been married about a year, and that her child was a girl; she moreover stated that she remembered nothing of what had taken place the day before, after her delivery, until the evening. The temperature was 37.2° C. The urine, as drawn with the catheter, was of a very dark color, but contained no albumen.

28th.—Complained of great pain in the head.

29th.—There was no evidence of milk in the breasts; the discharge from the uterus was very offensive, and there was tenderness over the abdomen on pressure. The vagina for the next four days was washed out sometimes with a weak solution of carbolic acid and sometimes with Condy's fluid.

30th.—The abdomen was still more tender on pressure; tongue clean; pulse 120; temperature 29.1° C. The urine continued of a high color, but contained no albumen.

July 1st.—Very much better. Asked to be allowed to take solid food, as she said she was very hungry.

From this date she steadily improved and left her bed on the 5th of July, only ten days after her labor.

The incision in the skin of the woman's right arm healed up directly; that in the left arm is not quite healed, but nearly so.

Mr. Cameron felt no inconvenience further than a feeling of want of energy for the following few days. A diffuse ecchymosis appeared on his forearm, but this was of no real moment.—*London Lancet.*

*On examining the instrument afterwards, it was ascertained that the india-rubber of which the injecting ball was made was cracked for a considerable portion of its circumference at its junction with the mountings. There can be no doubt that this was due to a defect in the india-rubber which had been overlooked. In other respects no instrument could have done its work better.

